# Prevalence and risk factors of frailty in patients with chronic obstructive pulmonary disease: systematic review and meta-analysis

**DOI:** 10.1007/s41999-023-00800-2

**Published:** 2023-07-12

**Authors:** Li-Cong Yan, Hong-Yan Lu, Xiao-Yan Wang, Gang Xiao, Yan Chang, Ping Yuan, Bei Wang

**Affiliations:** 1 Department of Respiratory and Critical Care Medicine, The General Hospital of Ningxia Medical University, Ningxia, 750004 China; 2 Department of Nursing, The General Hospital of Ningxia Medical University, No. 804, Shengli Street, Yinchuan City, 750004 Ningxia China; 3 College of Nursing, He Xi College, Zhangye, 734000 China; 4 Department of Radiology, Yinchuan Stomatology Hospital, Ningxia, 750000 China

**Keywords:** Chronic obstructive pulmonary disease, Frailty, Risk factors, Meta-analysis

## Abstract

**Objective:**

To systematically review the prevalence and risk factors for frailty in patients with chronic obstructive pulmonary disease (COPD).

**Methods:**

A systematic review and meta-analysis were conducted, and a search of the PubMed, Embase and Web of Science databases was carried out to collect Chinese and English studies on frailty and COPD published up to September 5, 2022.

**Results:**

A total of 38 articles were included for the quantitative analysis after the collected literature was either included or omitted based on pertinent criteria. The results indicated that the estimated overall pooled prevalence of frailty was 36% (95% confidence interval [CI] = 31–41%), and the estimated pre-frailty was 43% (95% CI = 37–49%). A higher age (odds ratio [OR] = 1.04; 95% CI = 1.01–1.06) and higher COPD assessment test (CAT) score (OR = 1.19; 95% CI = 1.12–1.27) were associated with a significantly increased likelihood of frailty in patients with COPD. However, a higher educational attainment (OR = 0.55; 95% CI = 0.43–0.69) and higher income (OR = 0.63; 95% CI = 0.45–0.88) were associated with a significantly reduced risk of frailty in patients with COPD. A total of 17 other risk factors for frailty were identified via qualitative synthesis.

**Conclusion:**

The incidence of frailty in patients with COPD is high, and there are many influencing factors.

**Supplementary Information:**

The online version contains supplementary material available at 10.1007/s41999-023-00800-2.

## Key summary points

**Aim:**

This study is the first to explore the risk factors of frailty in chronic obstructive pulmonary disease (COPD) patients.

**Findings:**

The estimated prevalence of frailty in patients with COPD was 36%.

**Message:**

Age, education level, income level and CAT score are risk factors for frailty in COPD patients.

**Supplementary Information:**

The online version contains supplementary material available at 10.1007/s41999-023-00800-2.

## Introduction

Frailty is a syndrome occurring in the older people that is defined as a state of increased vulnerability due to an age-related decline in function and reserves, which results in a decline in the ability to cope with daily or acute stressors [1, 2]. Once stressors strike, patients with frailty are likely to experience adverse consequences, such as prolonged hospital stays, decreased activity, occurrence of a disability attack, increased mortality and adverse drug reactions [3]. In addition, the presence of frailty is often observed in patients with chronic morbidities, and its prevalence is positively related to age [4–6]. Aging (in patients aged ≥ 65 years) and chronic diseases can promote health deterioration and increase the risk and severity of frailty [7–9].

Chronic obstructive pulmonary disease (COPD) has become a major challenge for public health worldwide. It is a common disease with high global morbidity and mortality rates [10], and is expected to rank fifth in terms of global disease burden and fourth in terms of mortality by 2030 [11]. According to current knowledge, COPD and frailty have certain overlapping risk factors, including aging, smoking, neuroendocrine abnormality, impaired immune system, chronic inflammation, lack of exercise and decreased exercise ability [5, 9]. Moreover, COPD is the most frequently studied chronic respiratory disease associated with frailty [12]. Compared to participants without COPD, patients with the disease have a higher likelihood of frailty [9]. In previous studies, the prevalence of frailty ranged from 9 to 28%, while the prevalence of pre-frailty ranged from 48 to 64%, depending on the setting [9]. Compared to patients without frailty, patients with both this condition and lung disease tend to have a worse prognosis, including a higher prevalence of hospitalization and readmission, prolonged hospital stay, disability and self-reported adverse endpoints [13].

As a dynamic state, frailty can be improved by a targeted treatment strategy; however, if left untreated, it can become worse, with a greater risk of adverse outcomes. Understanding the risk factors that contribute to frailty in patients with COPD is critical and could facilitate early identification of patients at high risk of frailty, as well as highlight new targets for interventions to prevent or improve the condition. To date, the associated risk factors of frailty in patients with COPD have not been systematically explored, thus hindering a reliable implementation of effective frailty interventions. Therefore, the present study quantitatively synthesizes the existing evidence on the prevalence and risk factors for frailty with COPD by performing a systematic review and meta-analysis, with the aim of highlighting areas for future research.

## Methods

The meta-analysis followed the Preferred Reporting Items for Systematic Reviews and Meta-Analyses Statement (PRISMA) guidelines. The protocol used for the present study is available from the International Prospective Register of Systematic Reviews (PROSPERO) database (https://www.crd.york.ac.uk/PROSPERO/).

### Literature and data acquisition

Three databases (PubMed, Embase and Web of Science) were independently searched by two authors (L.C.Y. and X.Y.W.) from database inception to September 5, 2022. The medical subject heading terms and free words associated with frailty and COPD were used as keywords (e.g., chronic obstructive lung disease, chronic obstructive pulmonary diseases, chronic obstructive airway disease, chronic obstructive pulmonary disease, airflow obstruction, chronic, airflow obstructions, chronic, chronic airflow obstructions, chronic airflow obstruction and frailties, frailness, frailty syndrome, debility, and debilities). The entries were searched independently by the two authors, and other potential records were manually obtained from the references contained in related reviews and articles.

### Inclusion and exclusion criteria

All observational studies investigating the associations between frailty and COPD were included. The inclusion criteria were as follows: (1) articles in English; (2) cross-sectional, case–control or cohort studies; (3) adult studies (≥ 18 years of age); and (4) articles reporting the prevalence rate data and prevalence rate or those that provide sufficient data for calculating the frailty prevalence rate in patients with COPD.

The exclusion criteria were as follows: (1) non-original articles (e.g., reviews, editorial letters or conference abstracts); (2) articles without an explicit definition of frailty; (3) articles with a single symptom used to define frailty; (4) articles with combined serious illness (e.g., human immunodeficiency virus); and (5) duplicates.

In addition, in the COPD frailty prevalence meta-analysis, only the frailty prevalence pertaining to COPD in the baseline data was reported due to the limitations of the original data.

### Study selection and data extraction

After storing all relevant records, the two reviewers independently screened the literature using endnote software (Clarivate Analytics, the United States). First, the duplicates were deleted before the articles were checked by title and summary and the full text that met the inclusion and exclusion criteria was finally determined. A third reviewer became involved in the case of any disagreement.

The two reviewers independently extracted variables from the included articles, which included the following: (1) the basic information (author, country, year of publication, study design, sample size and setting); (2) exposure variables (prevalence of frailty and pre-frailty); (3) sociodemographic variables (percentage of males, age); (4) and the frailty assessment scale. A third reviewer approved the final version if there was no dispute at the time.

### Related definitions

COPD: COPD is a common disease that can be prevented and treated, characterized by persistent respiratory symptoms and airflow limitations, usually associated with airway and/or alveolar abnormalities caused by significant exposure to harmful particles or gases [14].

Frailty: frailty is a medical syndrome with multiple causes, characterized by a decline in strength, endurance, and physiological function that increases an individual's progressive dependence and/or susceptibility to death [15].

Pre-frailty: pre-frailty refers to the intermediate state between health and frailty. Pre-frailty can be reversed to a healthy state, and some frailty states can also be reversed to pre-frailty [16].

### Methodological quality assessment

The methodological quality was evaluated by two investigators using the Joanna Briggs Institute's critical appraisal checklist, which is widely applied in previous reports on prevalence [17]. In total, this checklist includes nine items, and the total score is positively related to the quality of the included studies. The studies were classified as high quality (≤ 49%), medium quality (50–69%) or low quality (≥ 70%) according to the percentage of ‘no’ answers [17, 18].

### Statistical analysis

Data on the prevalence and impact of frailty in patients with COPD were extracted by two independent reviewers using Microsoft Excel. The meta-analysis was performed using Review Manager version 5.4 and Stata version 15.0 software. The *I*^2^ statistical test was used to assess the heterogeneity of the included studies. When there was heterogeneity in the study (*I*^2^ > 50% or *P* < 0.05), the results were combined using the random-effect model, with a fixed-effect model used in all other cases. Combined odds ratios (ORs) with a corresponding 95% confidence interval (CI) were calculated using a fixed-effect or random-effect model to compare the risk factors for the prevalence of frailty, with the significance determined using a *Z* test. When the *P* value was < 0.05, the results were considered statistically significant. Sensitivity analysis was performed by omitting individual studies and then comparing the *P* values of the combined ORs. When there was no substantial difference in the corresponding *P* value of the combined ORs (*P* < 0.05), the results were considered reliable. Publication bias was assessed using Begg’s test and funnel plot analysis [19, 20]. When the *P* value was < 0.05, the results were regarded as statistically significant.

## Results

### Search results

**Fig. 1:**
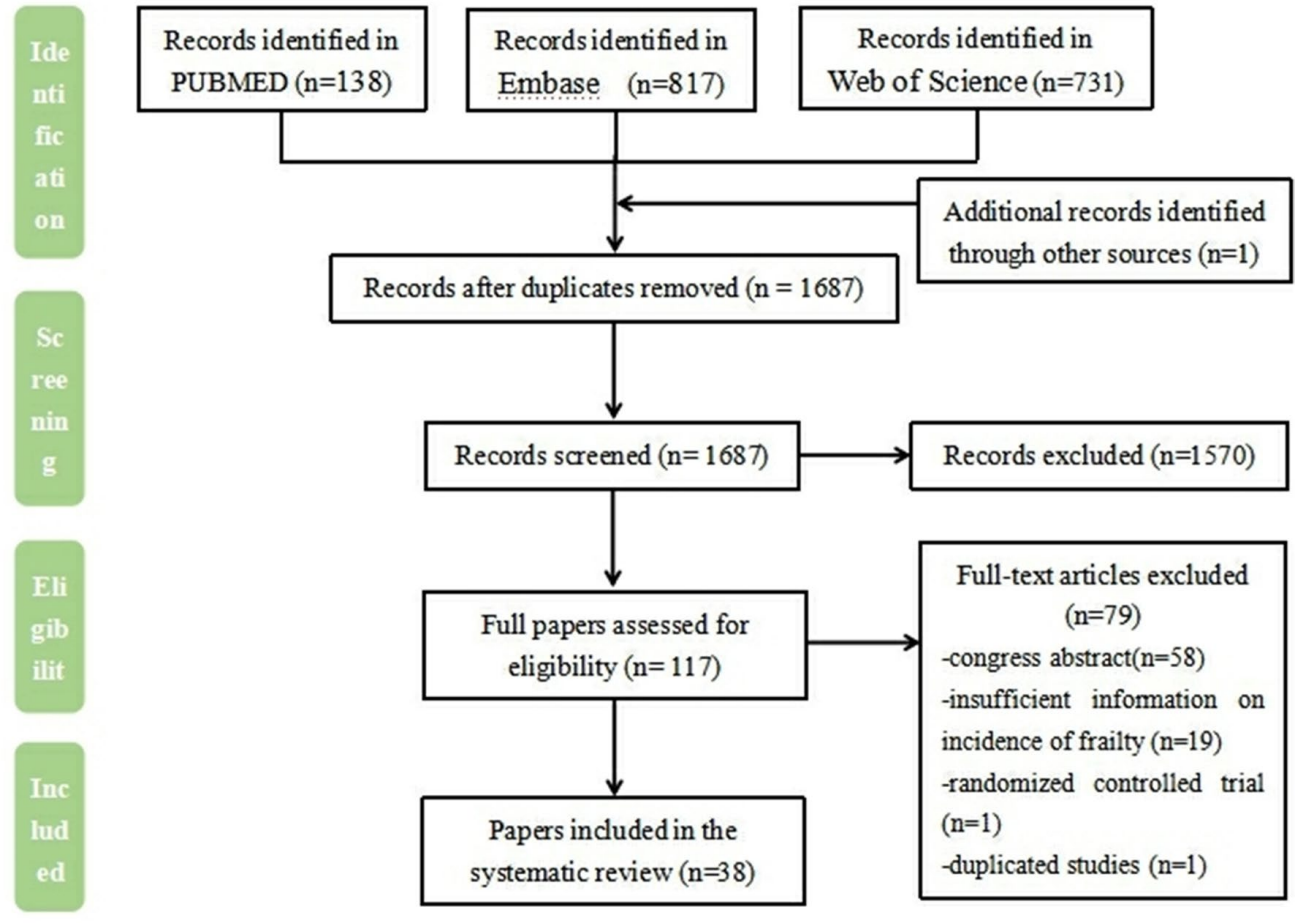
PRISMA flowchart

In total, 1687 relevant articles were identified from the PubMed (*n* = 138), Embase (*n* = 817) and Web of Science (*n* = 731) databases, with one article identified through other sources. After identifying any duplicate articles and any records marked as ineligible by the automation tools, 1570 articles were excluded. Following this, two reviewers screened the literature for highly relevant articles based on the titles and abstracts, with 117 considered for eligibility. After reviewing the full-text articles, 38 studies were finally determined to be eligible for the meta-analysis according to the inclusion criteria. The reasons for exclusion are detailed in Fig. 1.

### Data extraction

The characteristics of the included studies are presented in Table 1. These studies were published between 2011 and 2022, and 24 of them were cross-sectional studies [6, 7, 19, 21–41], 8 of which were longitudinal studies [17, 42–48] and the remaining 6 cohort studies [12, 49–53]. In total, 19,362 participants from 13 countries were included in the analysis, with the main geographic locations in the United States (eight studies) [28, 38, 40, 44–46, 49, 50]. The qualified studies involved 30 studies pertaining to hospitals. The mean age of the included participants ranged from 61 to 86 years, while in 31 of the studies, more than half of the participants were men. The prevalence of frailty in these studies ranged from 6 to 83%. The Fried frailty phenotype definition of frailty was used in 16 studies, the Kihon checklist was used in 4 studies [7, 24, 44, 47], the FRAIL scale was used in two studies [27, 37] and the frailty index was also used in two studies [4, 29].

**Table 1:** Characteristics of included studies

Author (first author/year study)	Design (study name)	County	Population type/setting	Sample size	Male%	Age/years (mean age ± SD)	Prevalence of frailty	Prevalence of pre-frailty	Frailty criteria	Quality
Hanlon (2022)	A longitudinal study	UK	UK Biobank participants Others	3132	54.90%	61.9 ± 5.9	16.41%	48.47%	Fried frailty phenotype (FFP)	High
Lee (2022)	A cohort study	Singapore	Community	1162	37.20%	68.3 ± 7.9	25.10%	–	Fried frailty phenotype (FFP)	High
Roberts (2022)	A cohort study	The United States	Community	4448	40.40%	–	22.54%	49.67%	Fried frailty phenotype (FFP)	High
Ushida (2022)	A cohort study	Japan	Hospital	3396	79.60%	75.9 ± 11.2	14.00%	–	Hospital frailty risk score (HFRS)	High
Zhang (2022)	A longitudinal study	China	Hospital	302	77.80%	86 (80–90)	51.00%	–	Fried frailty phenotype (FFP)	High
Bozkurt (2021)	A cross-sectional study	Turkey	Hospital	311 (≥ 65)	74.00%	71.7 ± 6.0	50.20%		Edmonton frailty scale (EFS)	High
Gephine (2021)	A cross-sectional study	French	–	44	68.00%	66 ± 8	43.18%		Fried frailty phenotype (FFP)	Moderate
Kagiali (2021)	A cross-sectional study	Turkey	Hospital	48	79.17%	–	41.67%		Fried frailty phenotype (FFP)	High
Luo (2021)	A cross-sectional study	China	Hospital	309	78.00%	86 (80, 90)	49.80%		Fried frailty phenotype (FFP)	High
Naval (2021)	A cross-sectional study	Spain	Hospital	127	85.00%	66.5 ± 7.9	24.40%	50.40%	Fried frailty phenotype (FFP)	High
Nishimura (2021)	A cross-sectional study	Japan	Hospital	89	93.26%	78 (74.0–82.0)	32.58%	25.84%	Kihon checklist (KCL)	High
Park (2021)	A cross-sectional study	Korea	Centers for disease control and prevention	417	98.10%	65.36 ± 9.35	35.50%		A new integral operational definition of frailty	High
Scarlata (2021)	A longitudinal study	The United States	Hospital	150	72.00%	73 ± 8	47.33%		Frailty index(FI)	High
Takahashi (2021)	A cross-sectional study	Japan	Hospital	40	97.50%	70.63 ± 8.21	50.00%	27.50%	Kihon checklist (KCL)	High
Witt (2021)	A cross-sectional study	The United States	Hospital	70	44.00%	63.5 (58.1, 71.3)	67.00%		Fried frailty phenotype (FFP)	High
Bernabeu-Mora (2020)	A longitudinal study	Spain	Hospital	119	87.40%	66.9 ± 7.9	7.6%	73.1%	Fried frailty phenotype (FFP)	High
Chin (2020)	A cross-sectional study	Canada	Hospital	50	–	72 ± 9 (mildly frail), 72 ± 10 (moderately frail), 76 ± 12 (severely frail and very severely frail)	58% (mildly frail 18%, moderately frail 36%, severely frail and very severely frail 4%)		Clinical Frailty Scale (CFS)	Moderate
Dias (2020)	A cross-sectional study	Brazil	Hospital	153	54.90%	70.0 (65.0–73.0) (pre-frail), 67.0 (61.0–71.5) (frail)	50.30%	35.30%	FRAIL scale	High
Oishi (2020)	A cross-sectional study	Japan	Hospital	128	91.20%	73 (69–78)	37.50%	39.00%	Kihon checklist (KCL)	High
Talay (2020)	A cross-sectional study	Turkey	Hospital	61	96.90%	–	63.90%		Tilburg frailty test (TFT)	High
Beek (2020)	A cross-sectional study	Netherlands	Hospital	57	49.00%	61.2 ± 8.7	(EFIP) 83%, (FFP) 28%	(EFIP) 4%, (FFP) 63%	Evaluative Frailty Index for Physical (EFIP); Fried frailty phenotype (FFP)	High
Yee (2020)	A longitudinal study	The United States	Hospital	280	80.00%	68	23.00%	62.00%	Frailty phenotype model; muscle frailty measured by handgrip strength (HGS)	High
Ehsani (2019)	A longitudinal study	The United States	Hospital	42	66.67%	71 ± 7 (pre-frail), 71 ± 8 (frail)	52.38%	33.33%	UEF assessment	High
Ierodiakonou (2019)	A cross-sectional study	Greece	Primary care facility	257	79.40%	65 ± 12.3	82.00%		The “Frail non-Disabled” (FiND) questionnaire	High
Kennedy (2019)	A cohort study	The United States	Hospital	902	59.50%	67 (63–70)	6.00%		Fried frailty phenotype (FFP)	High
Chen (2018)	A cross-sectional study	China	Hospital	125	100.00%	77.36 ± 10.26	56.80%		Chinese-Canadian study of health and aging clinical frailty scale	High
Gale (2018)	A cross-sectional study	UK	Hospital	520	51.92%	66.1 ± 7.6	27.5%		FI-CGA	High
Medina-Mirapeix (2018)	A cross-sectional study	Spain	Hospital	137	87.60%	66.9 ± 8.3	8.70%	73.70%	Fried frailty phenotype (FFP)	High
Bernabeu-Mora (2017)	A longitudinal study	Spain	Hospital	103	93.20%	71.0 ± 9.1	55.3% (mildly frail 19.4%, moderately frail 17.5%, severely frail and very severely frail 18.4%)		Reported Edmonton Frail scale (REFS)	High
Kusunose (2017)	A cross-sectional study	Japan	–	79	–	74.8 ± 6.3	21.50%	30.40%	Kihon checklist (KCL)	High
Limpawattana (2017)	A cross-sectional study	Thailand	Hospital	121	92.56%	73.5 ± 8.9 (frail group)	6.60%		FRAIL scale	High
Lahousse (2016)	A cohort study	Netherlands	Hospital	402	57.00%	75 ± 9	10.20%		Fried frailty phenotype (FFP)	High
Maddocks (2016)	A cohort study	UK	Hospital	816	59.30%	69.8 ± 9.7	25.60%		Fried frailty phenotype (FFP)	High
Medina-Mirapeix (2016)	A longitudinal study	Spain	Hospital	103	93.20%	71 ± 9.1	55.30%	18.40%	Reported Edmonton frail scale (REFS)	High
Mittal (2016)	A cross-sectional study	The United States	Hospital	120	44.00%	64 ± 9	18.00%	64.00%	Fried frailty phenotype (FFP)	High
Valenza (2016)	A cross-sectional study	Spain	Hospital	212	78.80%	71.4 ± 9.60 (COPD Exacerbation Group), 71.6 ± 8.36 (Stable COPD Group)	63.70%		Frailty index (FI)	High
Park (2013)	A cross-sectional study	The United States	Community	211	56.00%	70.65	57.80%	21.80%	NHANES questions and sociodemographic data	High
Galizia (2011)	A cross-sectional study	Italy	Hospital	489	54.80%	74.9 ± 6.3	48.90%		Frailty staging system (FSS)	High

### Prevalence of frailty in patients with chronic obstructive pulmonary disease

**Fig. 2:**
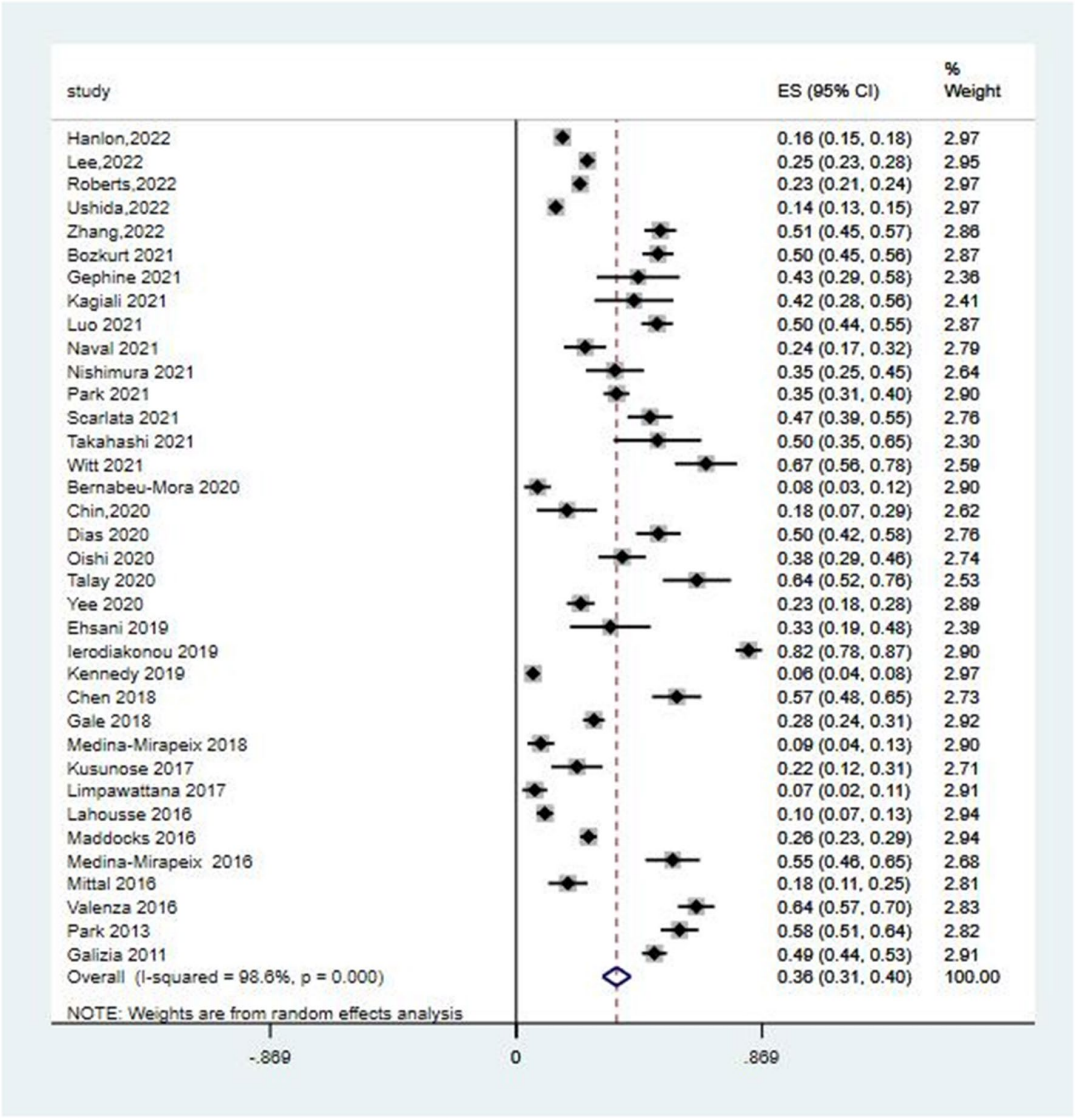
Forest plots of frailty in COPD

**Fig. 3:**
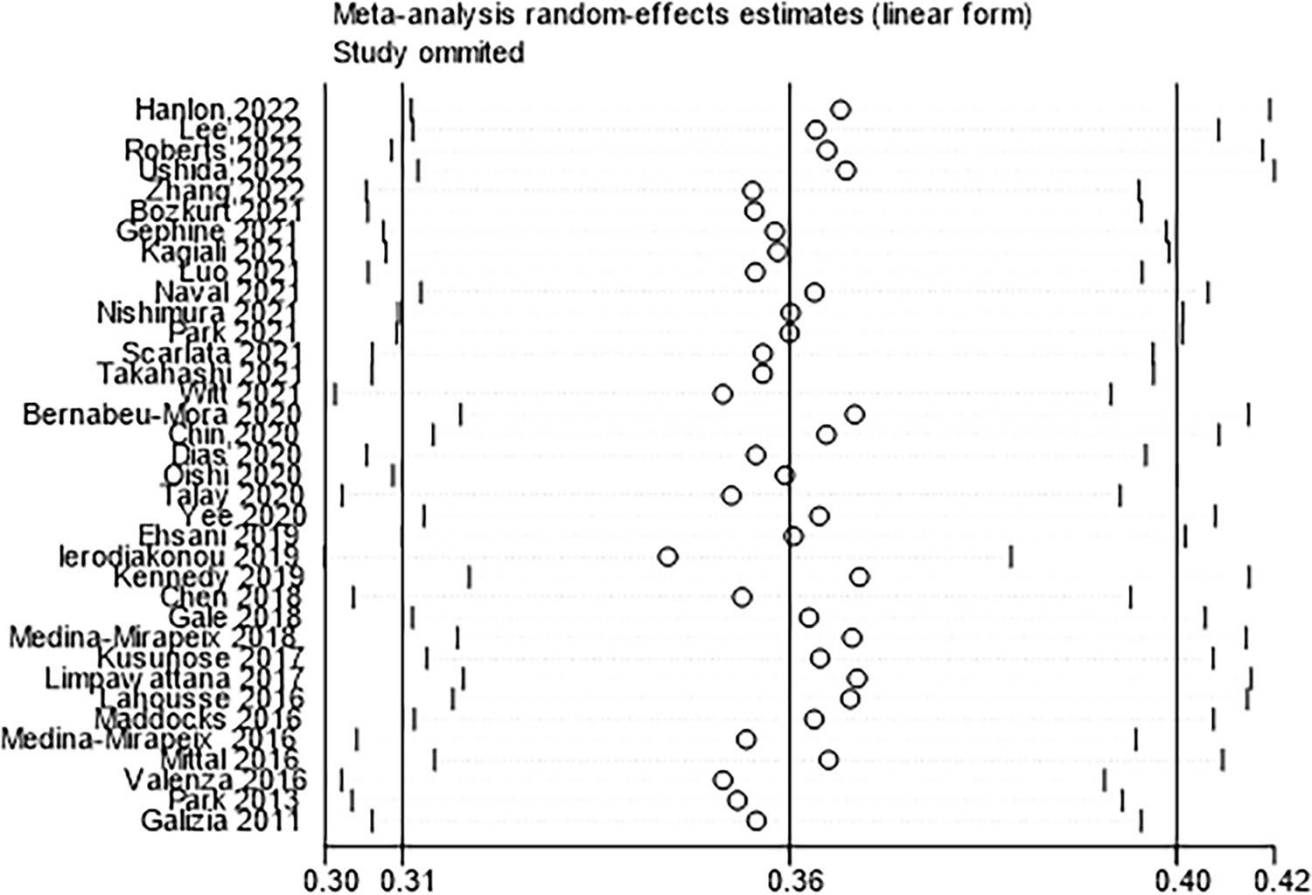
Sensitivity analyses

**Fig. 4:**
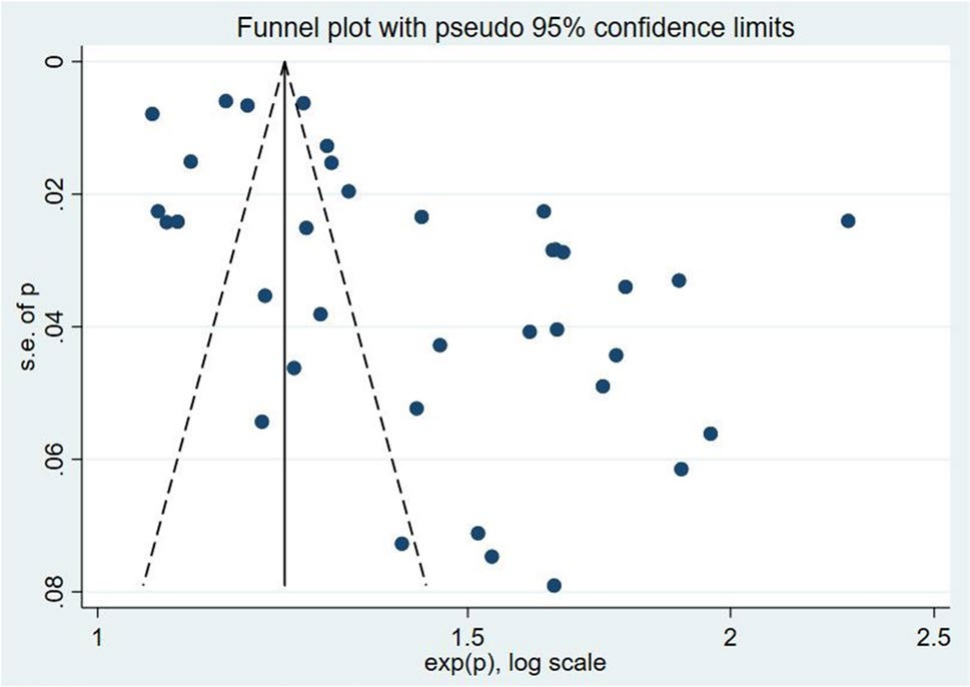
Funnel plot with pseudo 95% confidence limits

In total, 36 studies reported the prevalence of frailty in patients with COPD. The estimated overall pooled prevalence of frailty was 36% (95% CI = 31%–40%). There was significant heterogeneity (*I*^2^ = 98.6%, *P* < 0.001) among these studies (Fig. 2), and sensitivity analysis was thus conducted to explore the impact of a single study on the results of the meta-analysis. The analysis results indicated that the sensitivity was stable (Fig. 3). The funnel plot presented a largely asymmetric pattern (Fig. 4), which indicated the presence of publication bias.

### Prevalence of pre-frailty in patients with chronic obstructive pulmonary disease

**Fig. 5:**
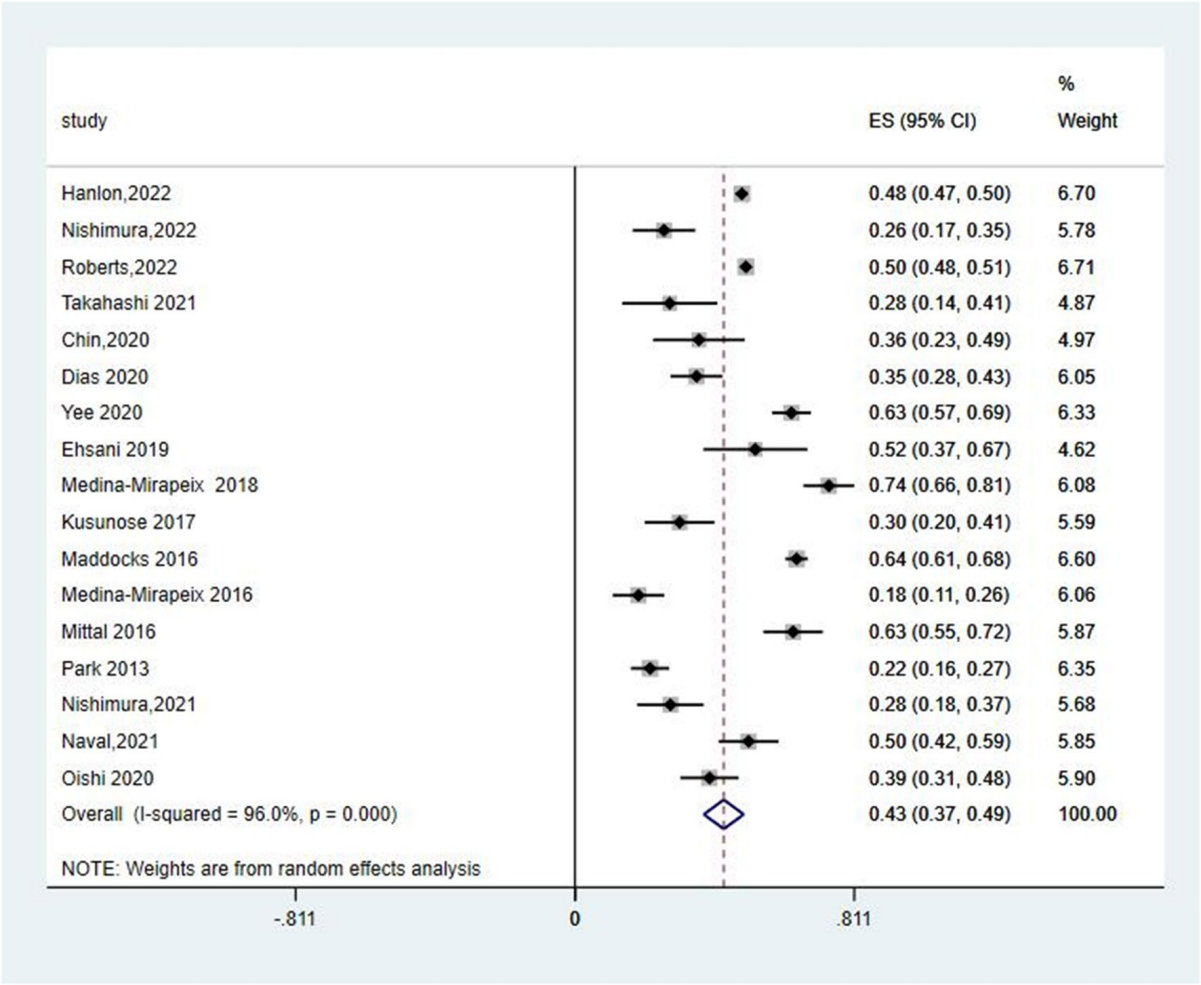
Forest plots of pre-frailty in COPD

Of the 38 included studies, only 17 reported the prevalence of pre-frailty in patients with COPD. The estimated overall pooled prevalence of pre-frailty was 43% (95% CI = 37–49%), with a high heterogeneity (*I*^2^ = 96.0%, *P* < 0.001) (Fig. 5).

### Meta-analysis of the risk factors for frailty in patients with chronic obstructive pulmonary disease

A total of six studies reported the risk factors for frailty in patients with COPD [24, 27, 32, 33, 37, 40]. Multivariate meta-analyses were performed in terms of nine risk factors (Table 2), with the most relevant detailed below.

**Table 2:** Risk factors of frailty in patients with COPD (univariate analysis)

Risk factor	No. of studies	Model of meta-analysis	OR (95% CI)	*P* value	Heterogeneity (*I*^2^) (%)
Age	5 [4, 7, 28, 39, 41]	Fixed	1.04 (1.02–1.06)	< 0.05	0
Education	3 [7, 28, 41]	Fixed	0.55 (0.43–0.69)	< 0.05	33
Income level	2 [7, 41]	Fixed	0.63 (0.45–0.88)	< 0.05	0
CAT score	2 [26, 41]	Fixed	1.19 (1.12–1.27)	< 0.05	0

#### Age

The meta-analysis of five studies [26, 32, 33, 37, 40] revealed that age was a risk factor for frailty in COPD (OR = 1.04; 95% CI = 1.01–1.06) (Supplemental File 1).

#### Educational attainment

The meta-analysis of three studies [26, 33, 40] revealed an association between educational attainment and the risk of frailty in patients with COPD. The results indicated that patients with a higher education were less likely to develop frailty (OR = 0.55; 95% CI = 0.43–0.69) (Supplemental File 2).

#### Income level

The meta-analysis of two studies [33, 40] revealed that a higher income (OR = 0.63; 95% CI = 0.45–0.88) was associated with a significantly reduced risk of frailty in patients with COPD (Supplemental File 3).

#### Assessment test score

A total of seven studies [17, 23, 24, 30, 31, 33, 35] investigated the relationship between the COPD CAT score and the risk of frailty in patients with COPD; however, most were unsuitable for extracting data and the meta-analysis could only be performed in terms of two of these studies [24, 33]. The results suggested that the CAT score was an indicator for frailty in patients with COPD (OR = 1.19; 95% CI = 1.12–1.27) (Supplemental File 4).

### Narrative synthesis of the other risk factors for frailty in patients with chronic obstructive pulmonary disease

In addition to the above factors, 17 studies examined the other risk factors for frailty in patients with COPD and identified 17 significant risk factors for the condition. Among these factors, two pertained to the sociodemographic domain, seven pertained to clinical performance, seven were health-related and one pertained to the social domain (Table 3). These studies results indicated that the number of exacerbations within a year (6 studies), comorbidities (5 studies), gender (5 studies), Global Chronic Obstructive Pulmonary Disease (GOLD) Symptom Initiative (4 studies), modified Medical Research Council (mMRC) score (3 studies), drug quantity (3 studies), smoking status (2 studies), and depression (2 studies) were risk factors for frailty in COPD patients. Inconsistent associations with frailty were observed for gender and body mass index (BMI). In one study, men were associated with a higher risk, while in four studies, women were associated with a higher risk. In one study, a higher BMI (> 26 kg/m^2^) was associated with a higher risk, while in another, a BMI of < 21 kg/m^2^ was associated with a higher risk. Previous frailty, depression, a high mMRC score, GOLD B&D status, active smoking status and a higher number of comorbidities, exacerbations and drugs used were consistently found to be correlated with an increased risk of frailty. Other risk factors were only investigated in a single study.

**Table 3:** Narrative synthesis of the others risk factors for frailty in patients with COPD (*n* = 17)

Factors	Factors
Sociodemographics	Health-related
Gender	Smoking status
Males [17] ↑	Current smoker [17, 39] ↑
Females [5, 7, 22, 28] ↑	Number of comorbidities [5, 17, 22, 42, 43] ↑
Marital status	Exacerbations [4, 19, 25, 26, 41, 42] ↑
Single or widower [22] ↑	Number of hospitalizations [25] ↑
Clinical	Number of drugs [17, 26, 42] ↑
BMI	Malnutrition [36] ↑
BMI < 21 [35] ↑	Depression [26, 30] ↑
BMI > 26 [17] ↑	Social factors
Poorer respiratory performance	Lower social support [17] ↑
FEV1% declines [25] ↑	
Higher mMRC score [5, 34, 43] ↑	
GOLD B&D status [5, 28, 34, 39] ↑	
Physical performance	
Higher 5STS test score [19] ↑	
6MWT meters declines [35] ↑	
Sarcopenia [4] ↑	

The predicted percentage of forced expiratory volume in 1 s (%FEV1)

The five-times sit-to-stand test (5STS)

The 6-min walking test (6MWT)

The modified Medical Research Council (mMRC) dyspnea scale

The COPD Assessment Test (CAT)

COPD patients were classified according to Global Initiative for Chronic Obstructive Lung Disease (GOLD) 2017 guidelines e.g., ABCD grading system. The ABCD grading system considers COPD health status, assessed by CAT or mMRC, along with exacerbation frequency and need for hospitalization (A is better, D is worse). We classified COPD patients to A-D groups based on both CAT and mMRC tools

Body mass index (BMI) was calculated as weight divided by height squared (Kg/m^2^)

## Discussion

The present study provides a comprehensive review of the existing evidence on frailty prevalence and risk factors in patients with COPD. The analysis indicated that the estimated frailty prevalence proportion among the patients with COPD was 36% (95% CI = 31–40%) and that the estimated pre-frailty prevalence proportion was 43% (95% CI = 37–49%).

The studies included in our meta-analysis largely report various sociodemographic variables as risk factors, and the results of the meta-analysis indicated that age, educational attainment, income level and CAT score were associated with a higher prevalence of frailty. In terms of the risk factors for frailty, a further 17 factors were found in the eligible studies. A prior study found that COPD and frailty share certain risk factors, including age and smoking [54, 55]. In fact, frailty is considered to be a geriatric syndrome [56], with its prevalence increasing with age. Older COPD patients are often accompanied by multiple diseases [57]. When multiple diseases coexist, various chronic diseases interact and have a synergistic effect on vascular endothelial cell damage, thereby exacerbating cerebral ischemia and hypoxia and forming a vicious cycle, further reducing cognitive function [57]. In addition, the coexistence of multiple diseases can accelerate the decline of organ function, leading to a decrease in physiological reserves of multiple systems. Patients are in a chronic state of depletion, resulting in a decrease in tolerance and resistance, leading to frailty [58]. Furthermore, in the case of smoking, systemic inflammation can be demonstrated by increased levels of inflammatory markers, such as high-sensitivity C-reactive protein and interleukin-6 [56, 59]. In fact, interleukin-6 is associated with sarcopenia, which is regarded as the main cause of physical frailty [59]. Most studies indicate that among patients with COPD, women are more prone to frailty than men, with research demonstrating that the bone mass loss caused by hormone-related changes associated with the menopause may be the mechanism behind the high vulnerability risk among older women [60].

The present study found that patients with COPD who had a poorer respiratory performance (e.g., first–second forced expiratory volume percentage declined, higher CAT and mMRC scores) and depression had a higher chance of developing frailty than those without these issues. It is well known that individuals with poor lung function have breathing difficulties and one possible reason for this is that individuals with dyspnea are more inclined to a sedentary lifestyle and are more likely to develop more muscle strength, mass and quality defects than other individuals [61, 62]. As the degree of difficulty in breathing increases, the patient's physical activity decreases, resulting in muscle atrophy and decreased muscle strength, which further leads to a decrease in activity tolerance and a vicious cycle. Exercise training is an important cornerstone in lung rehabilitation, as evidenced by multiple studies [63, 64] which have shown that reasonable exercise training can effectively improve exercise tolerance, delay muscle atrophy and muscle strength decline in COPD patients, and help delay or reverse the delay. In addition to respiratory symptoms and muscle frailty, patients with COPD may also suffer from anorexia and weight loss, which leads to malnutrition [9]. A study by Beek et al. indicated that malnutrition substantially (40%) coexists with frailty since energy and protein intake, as well as other key nutrients, play a fundamental role in muscle function and fatigue [19].

Furthermore, disease-related pain tends to increase the chances of patients with COPD taking multiple drugs [65, 66]. Changes associated with aging can affect specific pharmacokinetics, such as a decreased drug clearance and increased drug accumulation, while they may also increase the patient’s pain [67]. This may increase the risk of drug-related side effects, such as poor mobility and malnutrition [68] and could increase the risk of frailty. Therefore, it is recommended to carefully evaluate the risks of multiple drug use, optimize medication regimens for the older people, and reduce the damage caused by adverse drug reactions [15]. In the future, further exploration can be made to reduce the impact of multiple drug use on the development of frailty.

Finally, the prevalence of COPD is higher among populations with low socioeconomic status and low social support [69]. The present study revealed a high prevalence of frailty in patients with a low socioeconomic status (e.g., education and income) and social support. These social-network background aspects can be a factor in alleviating the defects accumulated in the older people over time by providing help in terms of daily activities, cognitive stimulation, love, friendship, suggestions for specific needs and/or financial and material goods or services. New insight into the prevention and the management of the onset of frailty could also be provided. However, it is unclear how these environmental factors affect the relationship between frailty and COPD and further research is needed to ascertain whether specific social factors can clarify this relationship.

The present study adopted a rigorous methodology following PRISMA guidelines and quantitatively synthesized the prevalence and risk factors for frailty with COPD, further expanding the knowledge in this field. This may help doctors make risk-informed decisions in clinical management.

However, the study involves a number of limitations. In general, the data pertaining to study-level variables, such as identification and vulnerability factors, tend to be inconsistent, which may have led to a certain degree of heterogeneity among the included studies, thus undermining the robustness of the results. In addition, the risk factors for frailty in patients with COPD were identified mainly based on limited studies and univariate analyses. These deficiencies were primarily due to the inconsistent risk factors reported in the original studies. Finally, due to the limited resources, the meta-analysis did not include any studies that were not published in English-language journals.

Furthermore, while strict retrieval strategies and inclusion and exclusion criteria were developed in this study, the following limitations must be acknowledged: (1) due to the lack of relevant research data, only the survival data from 38 original studies could be used for the meta-analysis, which may have reduced the statistical power of the study results; (2) the included literature was all from the PubMed, Embase or Web of Science databases, and all the relevant literature could not be collected; (3) the 38 included studies mostly used intermediate indicators (e.g., age, education level and income level) to observe the risk factors of frailty in patients with COPD and lacked specific hard-end points, such as the quality of the life score, mortality and other clinically relevant long-term follow-up outcomes, while they also did not determine the long-term risk factors.

## Conclusion

This systematic review and meta-analysis revealed that frailty substantially (36%) coexists with COPD. Older age, low socioeconomic status, poorer respiratory performance, a high number of comorbidities, exacerbations and drugs used, smoking, depression, poorer physical performance, malnutrition, a high number of hospitalizations and low social support are associated with the increased prevalence of frailty among patients with COPD. However, given the high heterogeneity among the included studies, which could have undermined the robustness of the results, further higher quality studies with a larger sample size must be conducted to validate the results.

## Supplementary Information

Below is the link to the electronic supplementary material.

Supplementary file1 (DOCX 81 KB)

Supplementary file2 (DOCX 72 KB)

Supplementary file3 (DOCX 68 KB)

Supplementary file4 (DOCX 68 KB)

## Data Availability

All data generated or analyzed during this study are included in this published article.
