# Supplementary figures and images for: Prevalence and risk factors of frailty in patients with chronic obstructive pulmonary disease: systematic review and meta-analysis
Source: Eur Geriatr Med. 2023 Jul 12;14(4):789–802. doi: 10.1007/s41999-023-00800-2 (PMC10447286; doi:10.1007/s41999-023-00800-2)

Supplemental File 1


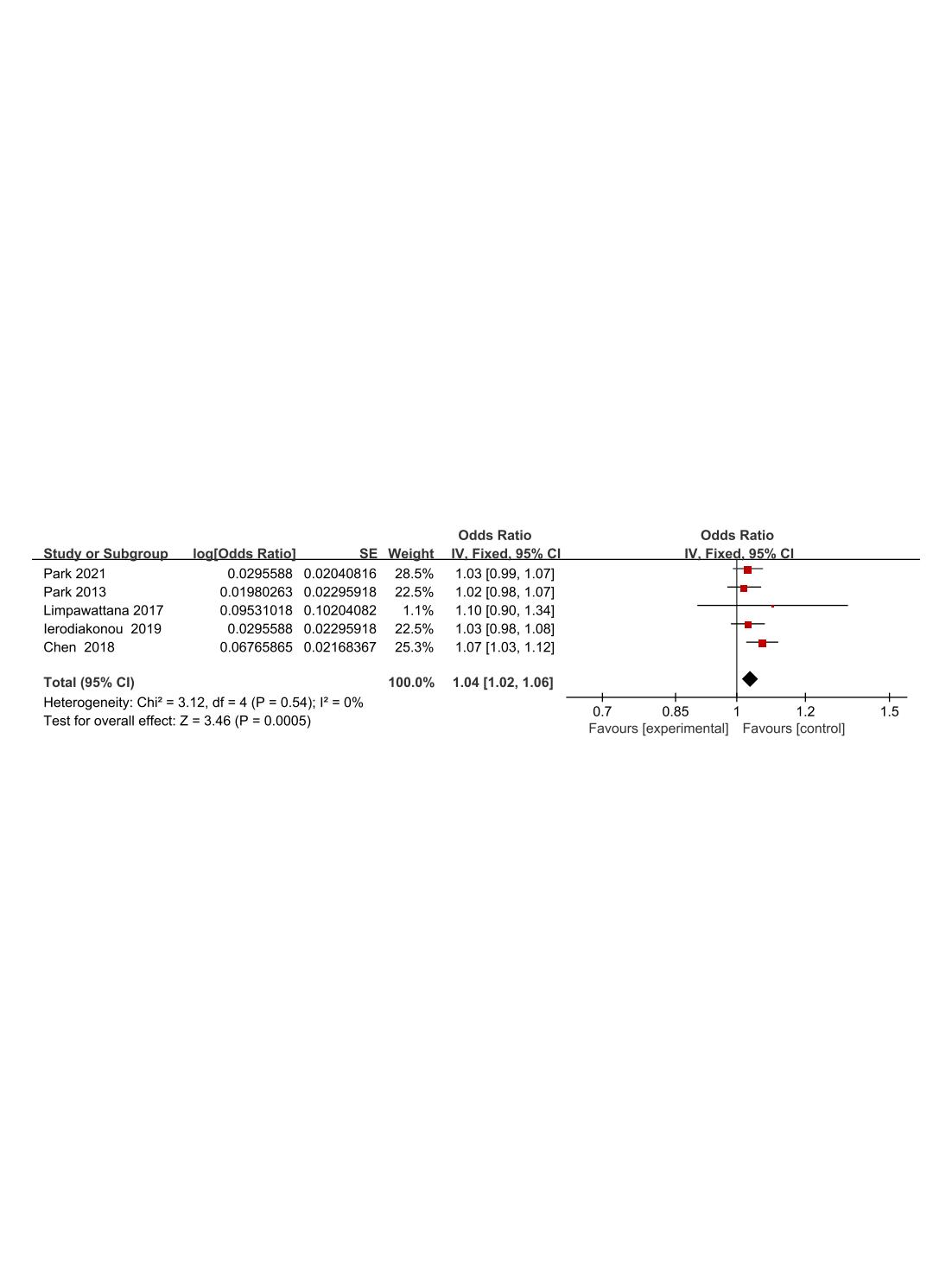

Supplement: Supplementary file 1 — Supplementary file1 (DOCX 81 KB) [file 41999_2023_800_MOESM1_ESM.docx]

Supplemental File 2


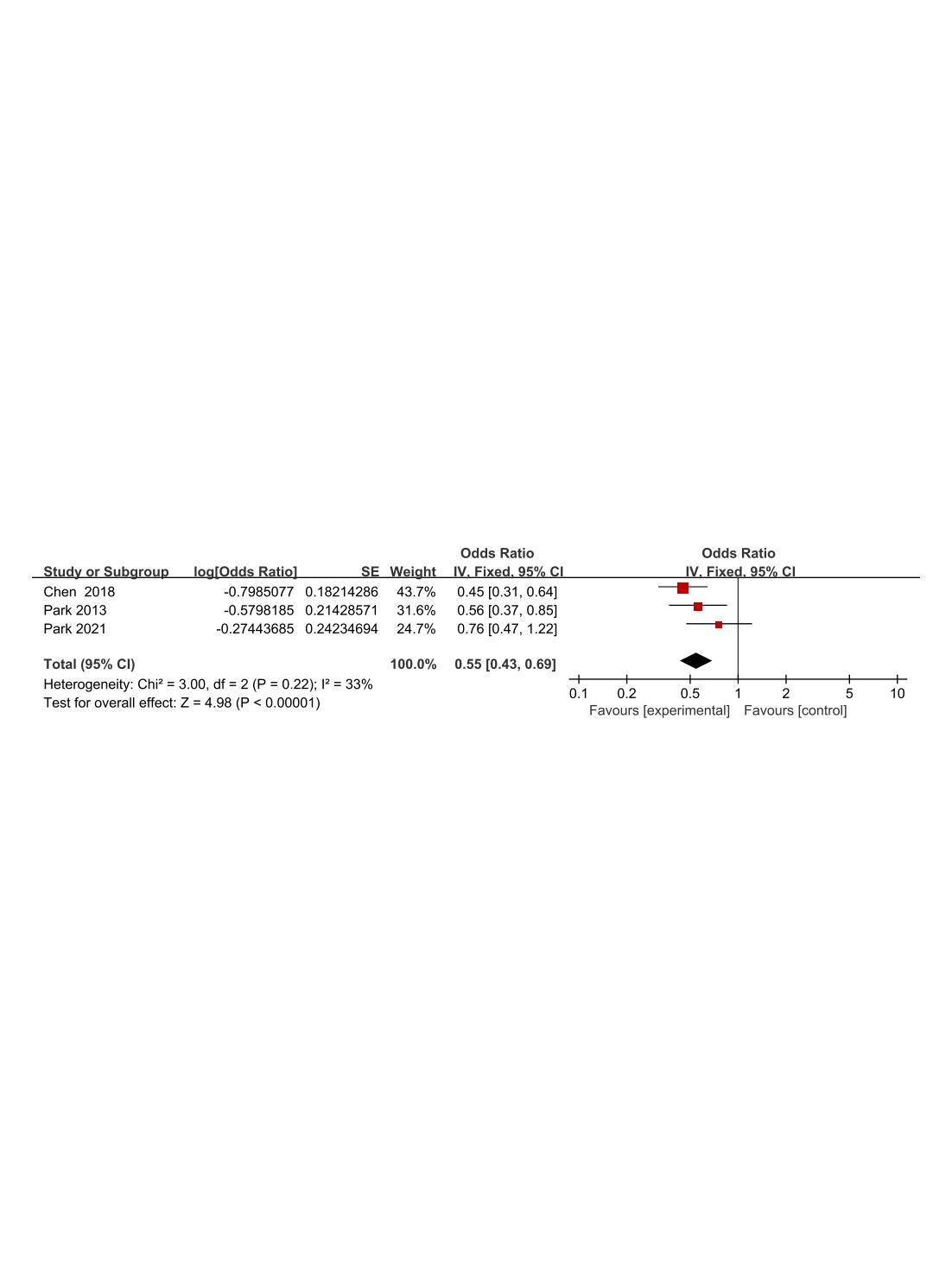

Supplement: Supplementary file 2 — Supplementary file2 (DOCX 72 KB) [file 41999_2023_800_MOESM2_ESM.docx]

Supplemental File 3


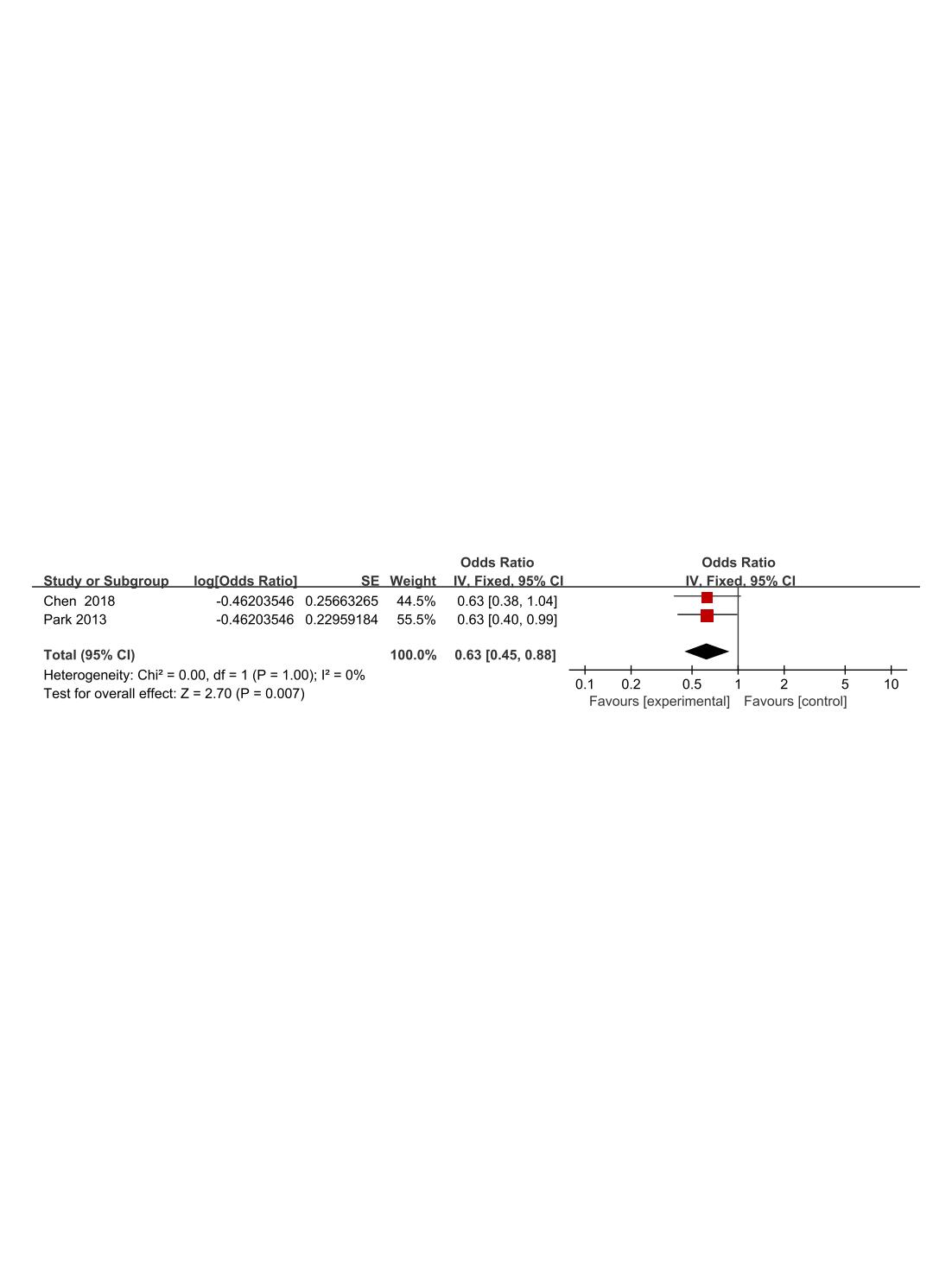

Supplement: Supplementary file 3 — Supplementary file3 (DOCX 68 KB) [file 41999_2023_800_MOESM3_ESM.docx]

Supplemental File 4


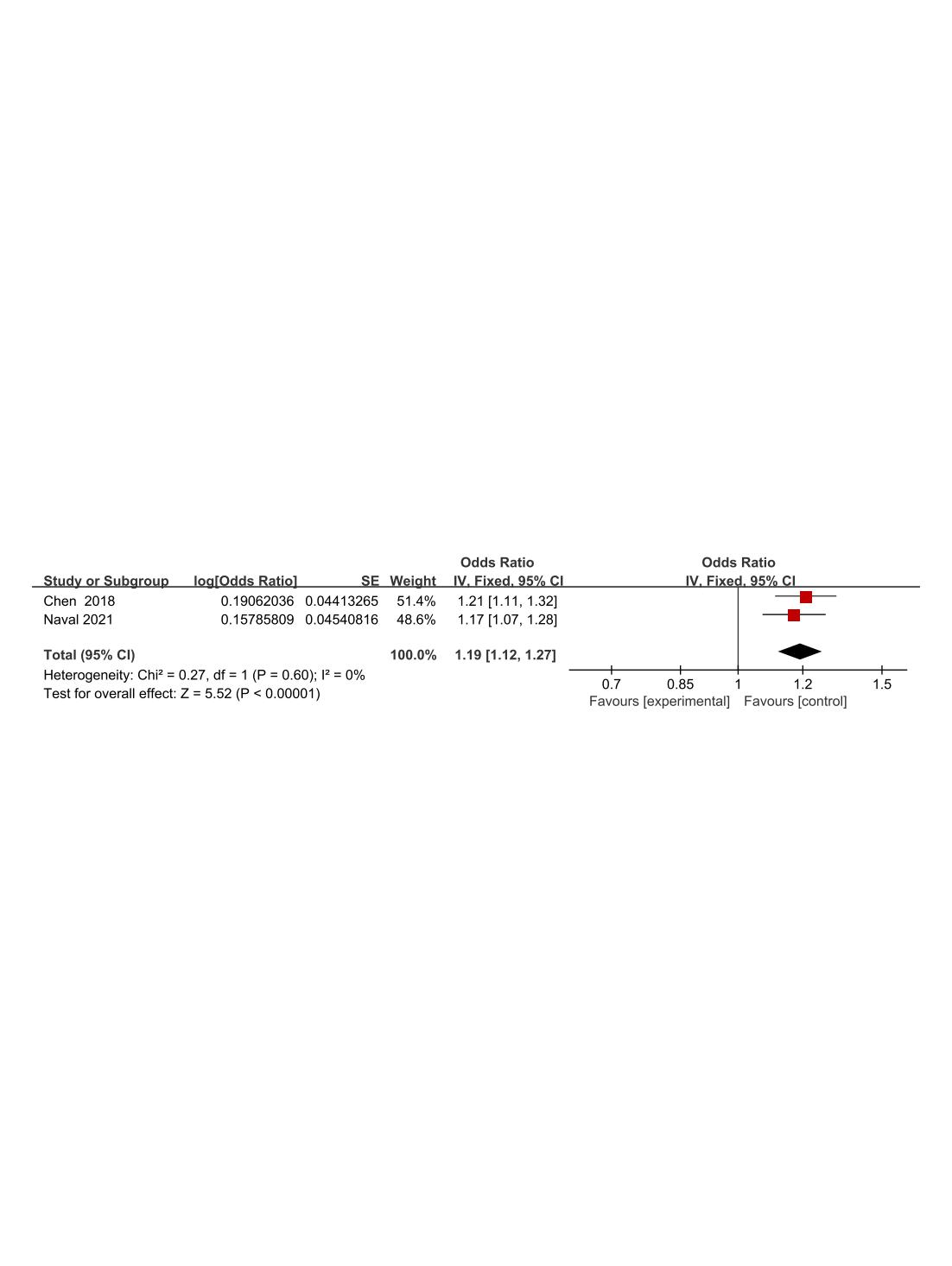

Supplement: Supplementary file 4 — Supplementary file4 (DOCX 68 KB) [file 41999_2023_800_MOESM4_ESM.docx]
